# Characterizing macular edema in retinitis pigmentosa through a combined structural and microvascular optical coherence tomography investigation

**DOI:** 10.1038/s41598-023-27994-6

**Published:** 2023-01-16

**Authors:** Alessandro Arrigo, Emanuela Aragona, Cristian Perra, Lorenzo Bianco, Alessio Antropoli, Andrea Saladino, Alessandro Berni, Giulia Basile, Adelaide Pina, Francesco Bandello, Maurizio Battaglia Parodi

**Affiliations:** 1 Department of Ophthalmology, IRCCS San Raffaele Scientific Institute, University Vita-Salute, Via Olgettina 60, 20132 Milan, Italy; 2 CNIT Research Unit, Department of Electrical and Electronic Engineering (DIEE), University of Cagliari, Cagliari, Italy

**Keywords:** Visual system, Biomarkers, Medical research

## Abstract

The aim of the study was to characterize macular edema (ME) in retinitis pigmentosa (RP) by means of quantitative optical coherence tomography (OCT)-based imaging. The study was designed as observational, prospective case series, with 1-year follow-up. All RP patients underwent complete ophthalmologic assessment, including structural OCT, OCT angiography, and microperimetry (MP). The primary outcome was the characterization through quantitative OCT-based imaging of RP eyes complicated by ME. A total of 68 RP patients’ eyes (68 patients) and 68 eyes of 68 healthy controls were recruited. Mean BCVA was 0.14 ± 0.17 LogMAR at baseline and 0.18 ± 0.23 LogMAR at 1-year follow-up (*p* > 0.05). Thirty-four eyes (17 patients; 25%) showed ME, with a mean ME duration of 8 ± 2 months. Most of the eyes were characterized by recurrent ME. The ME was mainly localized in the inner nuclear layer in all eyes. LogMAR BCVA was similar in all RP eyes, whether with or without ME, although those with ME were associated with higher vessel density values, as well as thicker choroidal layers, than those without ME. In conclusion, the inner retina is closely involved in the pathogenesis of ME. The impairment of retinal-choroidal exchanges and Müller cell disruption might be a major pathogenic factor leading to the onset of ME in RP.

## Introduction

Retinitis pigmentosa (RP) is one of the most common inherited retinal dystrophies, and is complicated by the onset of cystoid macular edema (ME) in approximatively 10–50% of cases^1,2^. The pathogenesis is still poorly understood and the current hypotheses involve the breakdown of the blood-retinal barrier (BRB), the dysfunction of retinal pigment epithelium (RPE) pumps, the impairment of macular Müller cells, as well as the presence of autoimmune-related phenomena and vitreous traction^3^. Current therapeutic options rely on topical or systemic carbonic anhydrase inhibitors, nonsteroidal anti-inflammatory drugs, steroids, anti-vascular endothelial growth factors and nutraceutical supplementations, with ME often assuming chronic or recurrent courses^3–6^. Multimodal imaging techniques now offer a more thorough, non-invasive way of investigating the characteristics and impairment of retinal and choroidal structures.

The main aim of the present study was to assess quantitative quantitative OCT-based imaging findings in a cohort of RP patients, to characterize the morphological features associated with the presence of ME and to investigate the clinical impact of ME on visual function and outcome.

## Materials and methods

The study was designed as a prospective, observational case series and included a 1-year follow-up. Patients affected by genetically confirmed RP were recruited in the Retinal Heredodystrophy Unit of the Department of Ophthalmology of IRCCS San Raffaele Scientific Institute (Milan, Italy). The study was approved by the ethical committee of IRCCS San Raffaele Scientific Institute (NET-2016-02363765), guaranteeing it would be carried out in accordance with the Declaration of Helsinki. Signed informed consent was obtained from all patients before they were included in the study.

The inclusion criteria were the genetically confirmed diagnosis of RP and age > 18 years. The exclusion criteria were macular atrophy or other causes of poor fixation, refractive errors greater than ± 3D, high media opacity, any other retinal and/or optic nerve diseases (e.g., diabetic retinopathy, glaucoma), any ophthalmic surgery in the last 6 months before inclusion in the study, and any systemic condition potentially affecting the results of the analyses. Furthermore, we carefully inspected the vitreoretinal interface imaging features to exclude RP eyes displaying an evident epiretinal membrane (ERM) or an incomplete posterior vitreous detachment, with vitreoretinal traction. We included only one randomly selected eye in the statistical analysis, for the entire cohort of RP patients. The "Results" section records only the prevalence of bilateral ME and the clinical course over the follow-up.

The ophthalmologic examination included best corrected visual acuity (BCVA) measurement, performed by means of the standard Early Treatment Diabetic Retinopathy Study (ETDRS) chart, slit-lamp anterior and posterior segment evaluation, and Goldmann applanation tonometry. RP eyes affected by ME were treated with topical or systemic drugs, at the ophthalmologists’ discretion.

Multimodal imaging included fundus blue autofluorescence (FAF), structural optical coherence tomography (OCT) (Spectralis HRA2 + OCT, Heidelberg Engineering; Heidelberg, Germany), and OCT angiography (OCTA) (Swept source DRI Triton Topcon, Topcon inc., Japan). Structural OCT image protocol included radial, raster and dense foveal scans with enhanced depth imaging (EDI). OCTA acquisitions included 4.5 × 4.5 mm and 12 × 12 mm foveal scans showing Topcon quality index > 70.

The quantitative assessment of structural OCT images included a global analysis and a detailed analysis performed through an ETDRS-9 sectors grid (central, inner nasal, inner superior, inner temporal, inner inferior, outer nasal, outer superior, outer temporal, outer inferior).

For structural OCT images we adopted the ETDRS-9 sectors grid already included in the Heidelberg software. In this case, the automatic segmentation tool was adopted under the supervision of two expert ophthalmologists (AA, EA), providing an ETDRS thickness map for the following layers: central macular thickness (CMT), retinal thickness (RT), retinal nerve fiber layer (RNFL), ganglion cell layer (GCL), inner plexiform layer (IPL), inner nuclear layer (INL), outer plexiform layer (OPL), outer nuclear layer (ONL), and ellipsoid zone thickness (EZ). The quantitative structural OCT evaluation also included choroidal thickness (CT) and Sattler and Haller layers (SLT and HLT, respectively), measured on a high-resolution, subfoveal, horizontal EDI structural OCT scan, and the calculation of the choroidal vascularity index (CVI). Choroidal thicknesses were manually measured by considering five single standardized measures performed beneath the fovea, at a distance of 750 µm and 1500 µm from the fovea, on the left- and right-hand sides, respectively. The mean measure was adopted for the analyses. CVI was calculated after binarizing the structural OCT reconstruction of the choroid. We then calculated the ratio between the black pixels (choroidal vessels) and white (stroma)^7^. To make it easier to interpret the structural OCT results, we considered the global values of the inner EDTRS grid and the mean values of the outer.

**Figure 1:**
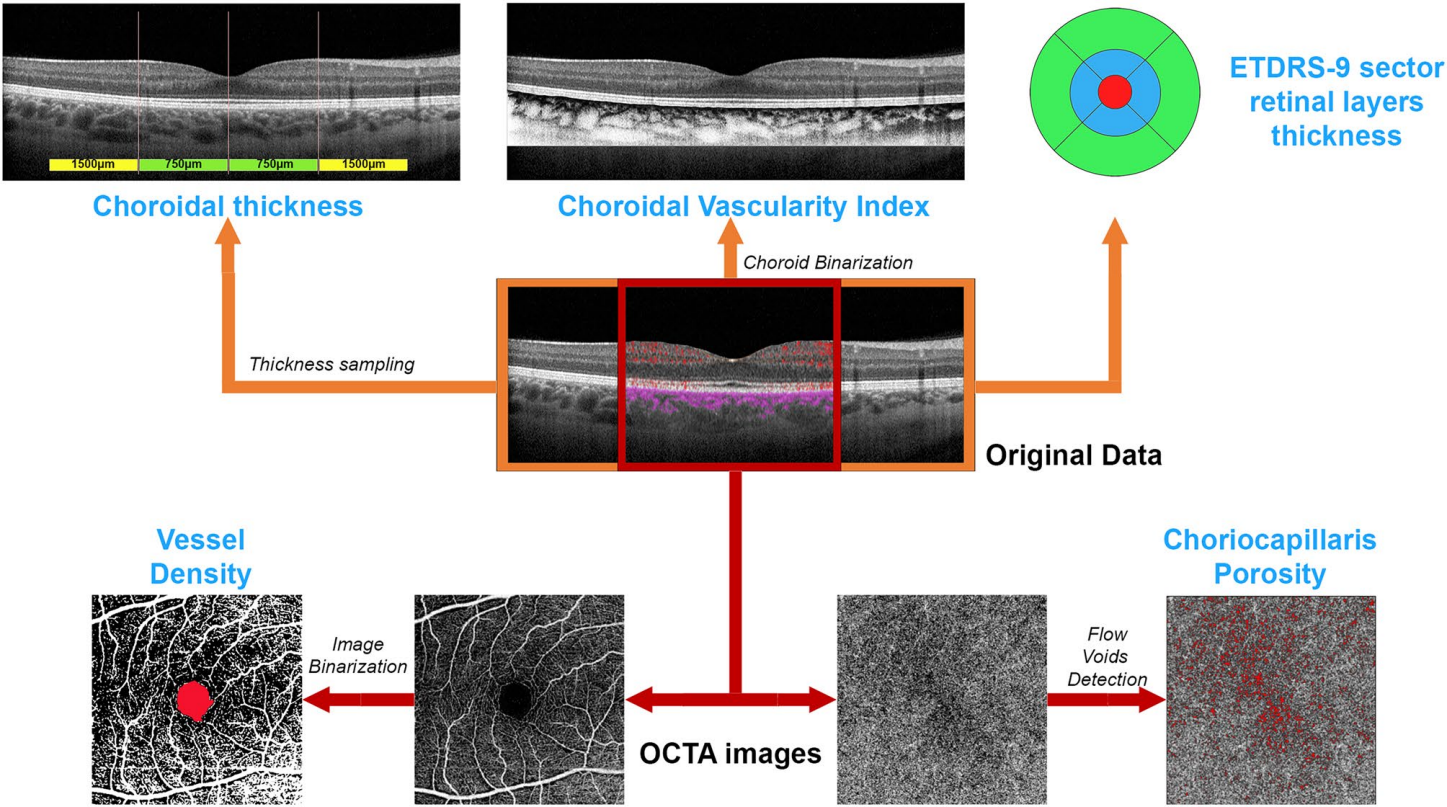
Quantitative analysis scheme. Starting from the original OCT and OCTA data, we obtained the thickness measurements of the choroidal layers by considering five fixed samplings, subfoveal, at 750 µm (right–left) and at 1500 µm (right–left) (upper left part of the scheme). Choroidal vascularity index was calculated after the segmentation and the binarization of the choroid (upper central part of the scheme). Moreover, we used the automatic ETDRS-9 sector grid measurements of retinal layers (upper right part of the scheme). With respect to OCTA, we obtained the enface reconstructions of vascular plexa (lower central part of the scheme). After the binarization of the images and the exclusion of the FAZ area, we calculated vessel density metric (lower left part of the scheme). In addition, choriocapillaris porosity was calculated after the careful detection of the choriocapillaris flow voids (highlighted in red) (lower right part of the scheme).

For OCTA analyses, we started with the automatic segmentation of the superficial capillary plexus (SCP), deep capillary plexus (DCP) and choriocapillaris (CC), carefully checked by two experts and manually modified (AA, EA), if necessary. All the images were loaded in the ImageJ software package and in-house scripts were used to calculate vessel density (VD)^8^. The first step was to binarize the images by using a mean threshold, with the manual segmentation of the foveal avascular zone, which was excluded from the calculation,then we calculated the ratio between white and black pixels in the binarized OCTA images^9,10^. VD was calculated on 4.5 × 4.5 mm scans to obtain the best compromise between the macular field of view and the high resolution of the images. We also calculated CC porosity (CCP), intended as a measure of the flow voids characterizing the CC, which was obtained by the porosity pipeline included in ImageJ^11^. We considered the structural OCT and OCTA images acquired at both baseline and after the 1-year follow-up. The quantitative analysis is schematically shown in Fig. 1.

The statistical analyses were performed using the SPSS software package (SPSS, Illinois, USA). Age and gender were considered fixed factors. The univariate models were checked by testing the normality distribution of each variable through frequency histograms and quantile–quantile plots. Continuous variables were described as mean ± standard deviation, whereas frequency and proportions were viewed as categorical variables. We included only one randomly selected eye for each patient. Continuous variables were analyzed by means of a two-tailed T test. Pearson correlation analysis was adopted to assess the relationships between all the variables considered. The Bonferroni approach was employed to address the multiple comparisons. Bearing in mind that seventeen different variables were included, we set an alfa value statistical significance threshold of < (0.05/17 = 0.003). To make the text and tables easier to read, we used statistically significant values of *p* < 0.05. We calculated the intraclass correlation coefficient (ICC) to assess the agreement between the two graders (overall 0.89; range 0.88–0.93). The main outcome measure was the quantitative quantitative OCT-based imaging characterization of ME in RP.

## Results

We included 68 eyes of 68 RP patients (32 males; mean age 42 ± 10 years) and 68 eyes of 68 healthy controls (34 males; mean age 41 ± 9 years; LogMAR BCVA 0.0 ± 0.0). Mean BCVA was 0.14 ± 0.17 LogMAR at baseline and 0.18 ± 0.23 LogMAR at 1-year follow-up (*p* > 0.05). The genetic profile of our RP cohort is shown in Table 1. Mean axial length was 23.46 mm ± 1.02 for RP eyes and 23.15 mm ± 0.96 for controls (*p* > 0.05).

**Table 1:** Genetic analysis in retinitis pigmentosa.

Genetic analysis in retinitis pigmentosa	
Gene	No. of patients
ABCA4	10
USH2A	16
PROM1	7
CYP4V2	3
NR2E3	3
PDE6A	2
RP1L1	3
RPGR	4
CNGA1	2
CNGB1	2
FSCN2	3
BBS1	2
RLBP1	3
MYO7A	2
CEPB90	2
EYE	2
EYS	2

**Figure 2:**
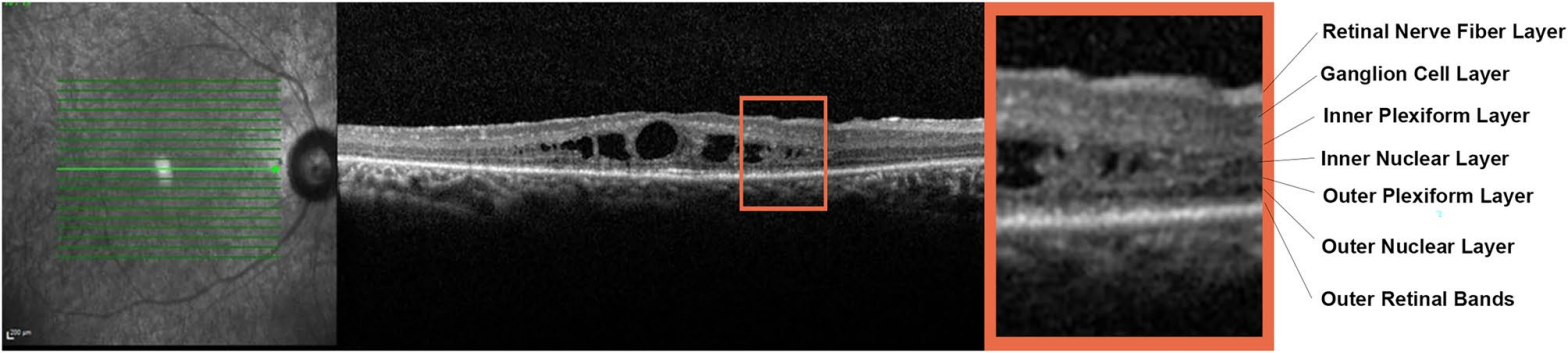
Macular edema localization in retinitis pigmentosa. The magnified image identifies each retinal layer and detects macular edema mainly localized in the inner nuclear layer.

In terms of prevalence of ME, 17 out of 68 patients (34 eyes; 25%) showed bilateral ME at the baseline visit (mean ME duration was 8 ± 2 months), whereas no further patients developed ME over the entire follow-up. ME was unsystematically managed with topical or systemic carbonic anhydrase inhibitors, or nonsteroidal anti-inflammatory drugs. No statistically relevant difference has been found looking at the treatments chosen with respect to the collected morpho-functional parameters. ME was found to have resolved in both eyes of 2 patients, whereas it was recurrent in 22 eyes (11 patients) and persistent in 8 eyes (4 patients). Structural OCT images revealed that INL was the main localization of ME in 100% of cases, with the IPL and OPL also being involved in the entire cohort of ME eyes (Fig. 2).

Complete clinical and imaging data are recorded in Table 2. CMT, CT and HLT values were similar in both RP patients and controls (all *p* > 0.05), whereas SLT values were found to be significantly lower in RP compared with healthy eyes (*p* < 0.05).

**Table 2:** Mean clinical and imaging data.

Clinical and imaging data			
	RP	Controls	*p* Value
No. of eyes	68	68	
Age	42 ± 10	41 ± 9	
Gender (M/F)	32/36	24/24	
LogMAR BCVA 0	0.14 ± 0.17	0.0 ± 0.0	< 0.05*
LogMAR BCVA 1Y	0.18 ± 0.23		< 0.05*
p Value	> 0.05		
CMT 0	284 ± 62	275 ± 21	> 0.05
CMT 1Y	278 ± 56		> 0.05
*p* Value	> 0.05		
CT 0	241 ± 112	258 ± 62	> 0.05
CT 1Y	237 ± 113		> 0.05
*p* Value	> 0.05		
SLT 0	56 ± 34	68 ± 21	< 0.05*
SLT 1Y	48 ± 33		< 0.05*
*p* Value	< 0.05*		
HLT 0	191 ± 81	204 ± 58	> 0.05
HLT 1Y	188 ± 83		> 0.05
*p* Value	> 0.05		

The following abbreviations are used: best corrected visual acuity (BCVA), central macular thickness (CMT), choroidal thickness (CT), Sattler layer thickness (SLT), Haller layer thickness (HLT). Statistically significant values, expressed for simplicity as *p* < 0.05, are marked by asterisks (*).

The quantitative analysis of RP eyes with and without ME is extensively shown in Table 3. LogMAR BCVA was similar in the two RP subgroups at both baseline and after the 1-year follow-up (*p* > 0.05). RP eyes with ME showed significantly higher SCP and DCP VD values, compared with RP eyes without ME (*p* < 0.05). On the other hand, CC VD, CCP and CVI were similar in all RP subgroups (*p* > 0.05). CT, HLT and SLT were found to be significantly higher in RP with ME, compared with RP without ME (*p* < 0.05). Moreover, the choroid resulted significantly thinner in RP eyes without ME than controls (all *p* < 0.05).

**Table 3:** Separate analysis of RP eyes affected by macular edema versus no macular edema. Only the statistically significant difference between RP eyes with and without macular edema are reported. The complete data are shown in Supplementary material.

Separated analysis macular edema vs no macular edema					
	RP with ME	RP without ME	Controls	*p* Value	
	1	2	3	1 versus 3	2 versus 3
VD_SCP					
Baseline	0.42 ± 0.02	0.39 ± 0.03	0.41 ± 0.01	> 0.05	< 0.05*
1-Year	0.42 ± 0.01	0.38 ± 0.03		> 0.05	< 0.05*
*p* Value 1 versus 2	< 0.05*				
VD_DCP					
Baseline	0.38 ± 0.02	0.33 ± 0.06	0.44 ± 0.01	< 0.05*	< 0.05*
1-Year	0.37 ± 0.02	0.33 ± 0.06		< 0.05*	< 0.05*
*p* Value 1 versus 2	< 0.05*				
CT					
Baseline	302 ± 142	227 ± 88	271 ± 67	> 0.05	< 0.05*
1-Year	321 ± 159	215 ± 81		> 0.05	< 0.05*
*p* Value 1 versus 2	< 0.05*				
HLT					
Baseline	228 ± 110	178 ± 71	203 ± 60	> 0.05	< 0.05*
1-Year	246 ± 123	173 ± 65		> 0.05	< 0.05*
*p* Value 1 versus 2	< 0.05*				
SLT					
Baseline	73 ± 34	49 ± 29	68 ± 23	> 0.05	< 0.05*
1-Year	75 ± 45	42 ± 26		> 0.05	< 0.05*
*p* Value 1 versus 2	< 0.05*				
Mean_RT_ALL					
Baseline	304 ± 26	273 ± 38	310 ± 15	> 0.05	< 0.05*
1-Year	306 ± 28	270 ± 38		> 0.05	< 0.05*
*p* Value 1 versus 2	< 0.05*				
Mean_RNFL_ALL					
Baseline	28 ± 7	23 ± 9	26 ± 3	> 0.05	< 0.05*
1-Year	26 ± 12	22 ± 9		> 0.05	< 0.05*
*p* Value 1 versus 2	< 0.05*				
Mean_GCL_ALL					
Baseline	36 ± 11	27 ± 12	40 ± 5	< 0.05*	< 0.05*
1-Year	34 ± 11	27 ± 12		< 0.05*	< 0.05*
*p* Value 1 versus 2	< 0.05*				
Mean_IPL_ALL					
Baseline	36 ± 7	29 ± 6	34 ± 3	> 0.05	< 0.05*
1-Year	35 ± 6	28 ± 6		> 0.05	< 0.05*
*p* Value 1 versus 2	< 0.05*				
Mean_INL_ALL					
Baseline	49 ± 2	38 ± 3	36 ± 3	< 0.05*	< 0.05*
1-Year	41 ± 3	38 ± 3		< 0.05*	< 0.05*
*p* Value 1 versus 2	< 0.05*				
Mean_OPL_ALL					
Baseline	37 ± 2	34 ± 4	30 ± 3	< 0.05*	< 0.05*
1-Year	40 ± 4	33 ± 5		< 0.05*	< 0.05*
*p* Value 1 versus 2	< 0.05*				
Mean_RT_INNER					
Baseline	335 ± 33	305 ± 42	340 ± 17	> 0.05	< 0.05*
1-Year	334 ± 34	300 ± 42		> 0.05	< 0.05*
*p* Value 1 versus 2	< 0.05*				
Mean_RNFL_INNER					
Baseline	29 ± 10	21 ± 8	22 ± 2	< 0.05*	> 0.05
1-Year	23 ± 12	20 ± 8		< 0.05*	> 0.05
*p* Value 1 versus 2	< 0.05*				
Mean_GCL_INNER					
Baseline	50 ± 19	35 ± 18	50 ± 6	> 0.05	< 0.05*
1-Year	46 ± 18	34 ± 18		> 0.05	< 0.05*
*p* Value 1 versus 2	< 0.05*				
Mean_IPL_INNER					
Baseline	43 ± 12	32 ± 10	41 ± 3	> 0.05	< 0.05*
1-Year	41 ± 12	32 ± 10		> 0.05	< 0.05*
*p* Value 1 versus 2	< 0.05*				
Mean_INL_INNER					
Baseline	58 ± 4	45 ± 5	41 ± 4	< 0.05*	< 0.05*
1-Year	49 ± 4	44 ± 5		< 0.05*	< 0.05*
*p* Value 1 versus 2	< 0.05*				
Mean_RT_OUTER					
Baseline	269 ± 20	242 ± 37	300 ± 15	< 0.05*	< 0.05*
1-Year	273 ± 24	240 ± 37		< 0.05*	< 0.05*
*p* Value 1 versus 2	< 0.05*				
Mean_RNFL_OUTER					
Baseline	30 ± 7	27 ± 13	34 ± 5	< 0.05*	< 0.05*
1-Year	28 ± 14	25 ± 12		< 0.05*	< 0.05*
*p* Value 1 versus 2	< 0.05*				
Mean_GCL_OUTER					
Baseline	25 ± 7	21 ± 10	36 ± 4	< 0.05*	< 0.05*
1-Year	23 ± 7	21 ± 10		< 0.05*	< 0.05*
*p* Value 1 versus 2	< 0.05*				
Mean_IPL_OUTER					
Baseline	30 ± 4	26 ± 5	30 ± 3	> 0.05	< 0.05*
1-Year	29 ± 3	25 ± 5		> 0.05	< 0.05*
*p* Value 1 versus 2	< 0.05*				
Mean_INL_OUTER					
Baseline	36 ± 2	31 ± 4	34 ± 3	> 0.05	< 0.05*
1-Year	34 ± 3	31 ± 6		> 0.05	< 0.05*
*p* Value 1 versus 2	< 0.05*				
Mean_OPL_OUTER					
Baseline	37 ± 2	31 ± 4	27 ± 2	< 0.05*	< 0.05*
1-Year	39 ± 5	31 ± 4		< 0.05*	< 0.05*
*p* Value 1 versus 2	< 0.05*				

The following abbreviations are used: choroidal thickness (CT), Sattler layer thickness (SLT), Haller layer thickness (HLT), vessel density (VD), superficial capillary plexus (SCP), deep capillary plexus (DCP), retinal thickness (RT), retinal nerve fiber layer (RNFL), ganglion cell layer (GCL), inner plexiform layer (IPL), inner nuclear layer (INL), outer plexiform layer (OPL). Statistically significant values, expressed for simplicity as p < 0.05, are marked by asterisks (*).

Looking at the structural OCT measurements, RNFL was thicker in RP eyes with ME in the inner ETDRS grid (*p* < 0.05), while the difference between RP eyes without ME and healthy controls was negligible in this regard (*p* > 0.05). GCL was thinner in RP overall than in healthy controls (*p* < 0.05). INL and OPL were always thicker in all RP eyes than in controls (*p* < 0.05), and even thicker in RP eyes with ME than RP eyes without (*p* < 0.05). No age effect was recorded in any of the tests regarding RP eyes either with or without ME. Moreover, axial length was similar in both RP subgroups (24.65 ± 1.52 for ME and 25.08 ± 1.44 for non-ME RP eyes, *p* > 0.05).

The correlation analysis revealed a significant positive relationship between CMT and both HLT and SLT (overall Pearson coeff. 0.416 and 0.383, respectively; *p* < 0.05). LogMAR BCVA significantly correlated with all VD metrics and CCP (overall Pearson coeff. − 0.457; *p* < 0.05).

No genotype–phenotype correlation was found considering all the quantitative parameters included in the analysis (*p* > 0.05).

## Discussion

The present study is devoted to the characterization of ME in RP through quantitative OCT-based imaging, while also investigating possible characteristics discriminating between eyes complicated by ME and eyes without ME. Our analysis revealed that RP eyes with ME featured significantly higher OCTA intraretinal vascular parameters than RP eyes without ME, a thicker choroid, and similar CC status. Interestingly, LogMAR BCVA was similar in the two RP subgroups. This finding supports the hypothesis that cystoid ME does not notably affect visual prognosis in RP, being more associated with the integrity of the RPE-photoreceptor complex^3,12,13^. Moreover, our results identified the INL as a more closely involved site. Indeed, the ME was found to be localized in the INL in 100% of our cases. RP eyes with ME were also characterized by significantly thicker RNFL, GCL, IPL and OPL than RP eyes without ME. INL is known to be a highly complex retinal structure, displaying the combined presence of several cytotypes, including bipolar, amacrine, Müller and horizontal cells^14,15^. It is worth noting that most Müller cells are localized in the INL, projecting the thick and thin Müller cells towards the inner and outer retinal structures^16^. As in the interpretation of the same phenomenon found in glaucoma, the thickening of the INL might be considered a sign of retinal distress or degeneration, with negative prognosis^17^. Our imaging-based data cannot provide definite conclusions regarding the correct interpretation of the RNFL, GCL and IPL thickness sparing that characterizes RP eyes with ME. We might hypothesize that these layers occurring in RP with ME are preserved, unlike in RP without ME. In contrast, the outer retinal status was similar in RP eyes with ME and RP eyes without ME.

Looking at the vascular compartment, our findings revealed significantly higher VD values of SCP and DCP in RP eyes with ME than in RP eyes without ME. The vascular involvement in RP with ME is a debated topic. It is worth noting that a previous paper reported no statistically significant differences in VD and FAZ area values between RP eyes with or without ME, thus suggesting that the pathogenesis of ME in RP differs from what is observed in other exudative diseases of which the vascular component is a major pathogenic source^18^. Conversely, another paper reported a significant effect of ME on the RP microvascular structure, especially looking to the FAZ area^19^. These previous findings suggest the need of further larger investigations to draw definite conclusions regarding the interaction between ME and the intraretinal vascular network. In addition, our study found that RP eyes with ME displayed changes of CC status that were not significant compared with RP eyes without ME, and a significantly thicker choroid. This finding was previously described in the cross-sectional investigation performed by Iovino and colleagues^20^, which also reported a decreased CVI in RP eyes with ME. In the present study, we found that CVI was significantly lower in RP eyes than in healthy controls, with no significant differences between RP eyes with or without ME. This means that the choroid might have a role in the pathogenesis of ME, although the insubstantial changes of CVI suggest other causative components than choroidal inflammation and congestion.

Overall, bearing in mind the above-mentioned pathogenic hypotheses for RP-related ME based on our data (BRB breakdown, RPE pump dysfunction, Müller cell impairment, autoimmune-related phenomena, and vitreous traction)^3^, we can rule out the vitreous contribution, since the presence of an ERM or vitreoretinal tractions were among our exclusion criteria. Although we found no clear signs of inflammation in RP eyes with ME, we cannot rule out all autoimmune-related phenomena. Hyperreflective foci have already been shown to increase in RP, and are associated with inflammatory and degenerative intraretinal phenomena occurring independently of the presence of ME^21^. With respect to the pathogenic hypothesis of RPE-related dysfunctions, we found similar morphological RPE status both in eyes with and without ME. However, the lack of any macroscopic damage to RPE detected on OCT means we cannot completely rule out RPE cells as contributing to the pathogenesis of ME, especially bearing in mind the possible loss of RPE pump function.

On the other hand, our findings corroborated the pathogenic hypothesis of Müller cell impairment and BRB breakdown. Indeed, Müller cells are known to play a key role in regulating intra/extracellular fluid distribution and macular edema^16,22^, and these cells are also central to the homeostatic maintenance of the BRB^16^. The involvement of Müller cells in RP has already been established,their increased activity has been associated with a protective role against neurodegeneration^23^. When Müller cells are activated, their genetic profile is altered and higher metabolic efforts are potentially needed to preserve retinal photoreceptors^24,25^. In this scenario, we can suppose that the increased activity of these cells might lead to progressive deterioration in function, which may be expressed in different ways, including the onset of ME and the degeneration of retinal photoreceptors. Regarding ME, we might hypothesize that the distress of retinal Müller cells causes the loss of fluid homeostasis and contributes to the impairment of the inner BRB, with progressive accumulation of fluid in the INL. In addition, Müller cell distress might also be associated with increased production of VEGF, providing trophic support for the SCP and DCP networks, whose sparing might be interpreted as a compensatory mechanism for the functional impairment of the choroid. Although intriguing, this is mere speculation and requires validating by future histologic studies.

We are aware that our study is potentially hampered by several drawbacks, chief of which is the relatively low number of eyes. We also realize RP is a chronic retinal dystrophy, meaning that a 1-year follow-up is too brief a period to draw definite conclusions. Furthermore, although we took several steps to guarantee high image quality, we are also mindful that all imaging techniques are prone to being encumbered by artifacts^26^. In addition, we speculated about the involvement of different retinal cytotypes and of the BRB in absence of histologic validations, which would be useful in coming to firmer conclusions. Moreover, although we excluded eyes affected by ERM or other kinds of vitreoretinal disorders, we acknowledge these eyes are subject to ME too, probably with their own pathogenesis. Further studies are therefore warranted to investigate the morpho-functional profiles of different subtypes of ME complicating RP.

In essence, our study focused on major inner retinal involvement in the pathogenesis of ME in RP. Many factors might contribute to the onset of ME in RP, including Müller cell impairment, BRB breakdown, loss of RPE function and choroidal alterations. Further studies are needed to define in detail the contribution of glial and neuronal retinal cytotypes in the pathogenesis of ME in RP and their relationship with the response to treatments.

## Supplementary Information

Supplementary Table 1.

## Data Availability

The datasets used and/or analysed during the current study available from the corresponding author on reasonable request.
