# Supplementary material for: Characterizing macular edema in retinitis pigmentosa through a combined structural and microvascular optical coherence tomography investigation
Source: Sci Rep. 2023 Jan 16;13:800. doi: 10.1038/s41598-023-27994-6 (PMC9842653; doi:10.1038/s41598-023-27994-6)
Supplement: Supplementary file 1 — Supplementary Table 1. [file 41598_2023_27994_MOESM1_ESM.docx]

**Supplementary Material**

**Supplementary table 1:** Separate analysis of RP eyes affected by macular edema vs no macular edema. This is the complete version of Table 3, reporting only the significant differences between RP eyes with and without macular edema. The following abbreviations are used: best corrected visual acuity (BCVA), central macular thickness (CMT), choroidal thickness (CT), Sattler layer thickness (SLT), Haller layer thickness (HLT), vessel density (VD), superficial capillary plexus (SCP), deep capillary plexus (DCP), choriocapillaris (CC), CC porosity (CCP), choroidal vascularity index (CVI). Statistically significant values, expressed for simplicity as p<0.05, are marked by asterisks (*).

| **Separate analysis: Macular Edema vs No Macular Edema** | | | | | | |
| --- | --- | --- | --- | --- | --- | --- |
|  | | **RP with ME** | **RP without ME** | **Controls** | **p Value** | |
|  |  | **1** | **2** | **3** | **1 vs 3** | **2 vs 3** |
| **LogMAR_BCVA** | **Baseline** | 0.15 ± 0.1 | 0.13 ± 0.1 | 0.0 ± 0.0 | <0.05* | <0.05* |
|  | **1-Year** | 0.20 ± 0.13 | 0.16 ± 0.25 |  | <0.05* | <0.05* |
|  | **p Value 1 vs 2** | >0.05 | |  |  |  |
| **VD_SCP** | **Baseline** | 0.42 ± 0.02 | 0.39 ± 0.03 | 0.41 ± 0.01 | >0.05 | <0.05* |
|  | **1-Year** | 0.42 ± 0.03 | 0.38 ± 0.03 |  | >0.05 | <0.05* |
|  | **p Value 1 vs 2** | <0.05* | |  |  |  |
| **VD_DCP** | **Baseline** | 0.38 ± 0.02 | 0.33 ± 0.06 | 0.44 ± 0.01 | <0.05* | <0.05* |
|  | **1-Year** | 0.37 ± 0.02 | 0.33 ± 0.06 |  | <0.05* | <0.05* |
|  | **p Value 1 vs 2** | <0.05* | |  |  |  |
| **VD_CC** | **Baseline** | 0.50 ± 0.02 | 0.49 ± 0.02 | 0.51 ± 0.01 | >0.05 | <0.05* |
|  | **1-Year** | 0.49 ± 0.02 | 0.48 ± 0.02 |  | <0.05* | <0.05* |
|  | **p Value 1 vs 2** | >0.05 | |  |  |  |
| **CVI** | **Baseline** | 0.48 ± 0.32 | 0.47 ± 0.34 | 0.66 ± 0.22 | <0.05* | <0.05* |
|  | **1-Year** | 0.46 ± 0.36 | 0.44 ± 0.38 |  | <0.05* | <0.05* |
|  | **p Value 1 vs 2** | >0.05 | |  |  |  |
| **CCP** | **Baseline** | 14.6 ± 5.2 | 15 ± 4.8 | 4.2 ± 0.5 | <0.05* | <0.05* |
|  | **1-Year** | 15.4 ± 5.5 | 16.1 ± 5.6 |  | <0.05* | <0.05* |
|  | **p Value 1 vs 2** | >0.05 | |  |  |  |
| **CT** | **Baseline** | 302 ± 142 | 227 ± 88 | 271 ± 67 | >0.05 | <0.05* |
|  | **1-Year** | 321 ± 159 | 215 ± 81 |  | >0.05 | <0.05* |
|  | **p Value 1 vs 2** | <0.05* | |  |  |  |
| **HLT** | **Baseline** | 228 ± 110 | 178 ± 71 | 203 ± 60 | >0.05 | <0.05* |
|  | **1-Year** | 246 ± 123 | 173 ± 65 |  | >0.05 | <0.05* |
|  | **p Value 1 vs 2** | <0.05* | |  |  |  |
| **SLT** | **Baseline** | 73 ± 34 | 49 ± 29 | 68 ± 23 | >0.05 | <0.05* |
|  | **1-Year** | 75 ± 45 | 42 ± 26 |  | >0.05 | <0.05* |
|  | **p Value 1 vs 2** | <0.05* | |  |  |  |
| **Mean_RT_ALL** | **Baseline** | 304 ± 26 | 273 ± 38 | 310 ± 15 | >0.05 | <0.05* |
|  | **1-Year** | 306 ± 28 | 270 ± 38 |  | >0.05 | <0.05* |
|  | **p Value 1 vs 2** | <0.05* | |  |  |  |
| **Mean_RNFL_ALL** | **Baseline** | 28 ± 7 | 23 ± 9 | 26 ± 3 | >0.05 | <0.05* |
|  | **1-Year** | 26 ± 12 | 22 ± 9 |  | >0.05 | <0.05* |
|  | **p Value 1 vs 2** | <0.05* | |  |  |  |
| **Mean_GCL_ALL** | **Baseline** | 36 ± 11 | 27 ± 12 | 40 ± 5 | <0.05* | <0.05* |
|  | **1-Year** | 34 ± 11 | 27 ± 12 |  | <0.05* | <0.05* |
|  | **p Value 1 vs 2** | <0.05* | |  |  |  |
| **Mean_IPL_ALL** | **Baseline** | 36 ± 7 | 29 ± 6 | 34 ± 3 | >0.05 | <0.05* |
|  | **1-Year** | 35 ± 6 | 28 ± 6 |  | >0.05 | <0.05* |
|  | **p Value 1 vs 2** | <0.05* | |  |  |  |
| **Mean_INL_ALL** | **Baseline** | 49 ± 2 | 38 ± 3 | 36 ± 3 | <0.05* | <0.05* |
|  | **1-Year** | 41 ± 3 | 38 ± 3 |  | <0.05* | <0.05* |
|  | **p Value 1 vs 2** | <0.05* | |  |  |  |
| **Mean_OPL_ALL** | **Baseline** | 37 ± 2 | 34 ± 4 | 30 ± 3 | <0.05* | <0.05* |
|  | **1-Year** | 40 ± 4 | 33 ± 5 |  | <0.05* | <0.05* |
|  | **p Value 1 vs 2** | <0.05* | |  |  |  |
| **Mean_ONL_ALL** | **Baseline** | 49 ± 9 | 49 ± 12 | 70 ± 3 | <0.05* | <0.05* |
|  | **1-Year** | 49 ± 8 | 48 ± 11 |  | <0.05* | <0.05* |
|  | **p Value 1 vs 2** | >0.05 | |  |  |  |
| **Mean_EZ_ALL** | **Baseline** | 13 ± 3 | 14 ± 3 | 21 ± 2 | <0.05* | <0.05* |
|  | **1-Year** | 12 ± 3 | 12 ± 3 |  | <0.05* | <0.05* |
|  | **p Value 1 vs 2** | >0.05 | |  |  |  |
| **Mean_RT_INNER** | **Baseline** | 335 ± 33 | 305 ± 42 | 340 ± 17 | >0.05 | <0.05* |
|  | **1-Year** | 334 ± 34 | 300 ± 42 |  | >0.05 | <0.05* |
|  | **p Value 1 vs 2** | <0.05* | |  |  |  |
| **Mean_RNFL_INNER** | **Baseline** | 29 ± 10 | 21 ± 8 | 22 ± 2 | <0.05* | >0.05 |
|  | **1-Year** | 23 ± 12 | 20 ± 8 |  | <0.05* | >0.05 |
|  | **p Value 1 vs 2** | <0.05* | |  |  |  |
| **Mean_GCL_INNER** | **Baseline** | 50 ± 19 | 35 ± 18 | 50 ± 6 | >0.05 | <0.05* |
|  | **1-Year** | 46 ± 18 | 34 ± 18 |  | >0.05 | <0.05* |
|  | **p Value 1 vs 2** | <0.05* | |  |  |  |
| **Mean_IPL_INNER** | **Baseline** | 43 ± 12 | 32 ± 10 | 41 ± 3 | >0.05 | <0.05* |
|  | **1-Year** | 41 ± 12 | 32 ± 10 |  | >0.05 | <0.05* |
|  | **p Value 1 vs 2** | <0.05* | |  |  |  |
| **Mean_INL_INNER** | **Baseline** | 58 ± 4 | 45 ± 5 | 41 ± 4 | <0.05* | <0.05* |
|  | **1-Year** | 49 ± 4 | 44 ± 5 |  | <0.05* | <0.05* |
|  | **p Value 1 vs 2** | <0.05* | |  |  |  |
| **Mean_OPL_INNER** | **Baseline** | 39 ± 6 | 37 ± 5 | 34 ± 14 | <0.05* | <0.05* |
|  | **1-Year** | 43 ± 6 | 37 ± 6 |  | <0.05* | <0.05* |
|  | **p Value 1 vs 2** | >0.05 | |  |  |  |
| **Mean_ONL_INNER** | **Baseline** | 53 ± 14 | 55 ± 14 | 72 ± 4 | <0.05* | <0.05* |
|  | **1-Year** | 53 ± 13 | 54 ± 13 |  | <0.05* | <0.05* |
|  | **p Value 1 vs 2** | >0.05 | |  |  |  |
| **Mean_EZ_INNER** | **Baseline** | 14 ± 3 | 14 ± 2 | 22 ± 2 | <0.05* | <0.05* |
|  | **1-Year** | 14 ± 3 | 13 ± 3 |  | <0.05* | <0.05* |
|  | **p Value 1 vs 2** | >0.05 | |  |  |  |
| **Mean_RT_OUTER** | **Baseline** | 269 ± 20 | 242 ± 37 | 300 ± 15 | <0.05* | <0.05* |
|  | **1-Year** | 273 ± 24 | 240 ± 37 |  | <0.05* | <0.05* |
|  | **p Value 1 vs 2** | <0.05* | |  |  |  |
| **Mean_RNFL_OUTER** | **Baseline** | 30 ± 7 | 27 ± 13 | 34 ± 5 | <0.05* | <0.05* |
|  | **1-Year** | 28 ± 14 | 25 ± 12 |  | <0.05* | <0.05* |
|  | **p Value 1 vs 2** | <0.05* | |  |  |  |
| **Mean_GCL_OUTER** | **Baseline** | 25 ± 7 | 21 ± 10 | 36 ± 4 | <0.05* | <0.05* |
|  | **1-Year** | 23 ± 7 | 21 ± 10 |  | <0.05* | <0.05* |
|  | **p Value 1 vs 2** | <0.05* | |  |  |  |
| **Mean_IPL_OUTER** | **Baseline** | 30 ± 4 | 26 ± 5 | 30 ± 3 | >0.05 | <0.05* |
|  | **1-Year** | 29 ± 3 | 25 ± 5 |  | >0.05 | <0.05* |
|  | **p Value 1 vs 2** | <0.05* | |  |  |  |
| **Mean_INL_OUTER** | **Baseline** | 36 ± 2 | 31 ± 4 | 34 ± 3 | >0.05 | <0.05* |
|  | **1-Year** | 34 ± 3 | 31 ± 6 |  | >0.05 | <0.05* |
|  | **p Value 1 vs 2** | <0.05* | |  |  |  |
| **Mean_OPL_OUTER** | **Baseline** | 37 ± 2 | 31 ± 4 | 27 ± 2 | <0.05* | <0.05* |
|  | **1-Year** | 39 ± 5 | 31 ± 4 |  | <0.05* | <0.05* |
|  | **p Value 1 vs 2** | <0.05* | |  |  |  |
| **Mean_ONL_OUTER** | **Baseline** | 33 ± 5 | 33 ± 9 | 62 ± 4 | <0.05* | <0.05* |
|  | **1-Year** | 35 ± 6 | 33 ± 9 |  | <0.05* | <0.05* |
|  | **p Value 1 vs 2** | >0.05 | |  |  |  |
| **Mean_EZ_OUTER** | **Baseline** | 11 ± 7 | 12 ± 8 | 18 ± 2 | <0.05* | <0.05* |
|  | **1-Year** | 10 ± 8 | 9 ± 8 |  | <0.05* | <0.05* |
|  | **p Value 1 vs 2** | >0.05 | |  |  |  |
